# A Clinical Case of Polymyositis Complicated by Antisynthetase Syndrome

**DOI:** 10.7759/cureus.12737

**Published:** 2021-01-16

**Authors:** Aadil M Khan, Faim Ahmad, Usama Rehman, Himanshu Jindal, Hridya Harimohan

**Affiliations:** 1 Internal Medicine, Ganesh Shankar Vidyarthi Memorial Medical College, Kanpur, IND; 2 Anesthesia, Mayo Hospital, Lahore, PAK; 3 General Medicine, Ganesh Shankar Vidyarthi Memorial Medical College and Lala Lajpat Rai Hospital, Kanpur, IND; 4 Internal Medicine, Kerala Institute of Medical Sciences, Trivandrum, IND

**Keywords:** antisynthetase syndrome, interstitial lung disease, myositis, aminoacyl trna synthetase, anti-jo-1 antibodies

## Abstract

Antisynthetase syndrome is an autoimmune condition that manifests clinically through signs and symptoms, such as interstitial lung disease, myositis, Raynaud’s phenomenon, fever, hyperkeratotic fingertips (mechanic’s hands), and arthritis. It is associated with antibodies against aminoacyl tRNA synthetase enzyme, the most common autoantibody being the anti-Jo-1. An 18-year-old girl presented with weakness of both the upper and lower limb, swelling and generalized body pain, difficulty in swallowing. MRI of the thigh was highly suggestive of myositis with symmetrical bilateral involvement. Based on proximal muscle weakness, elevated creatine phosphokinase (CPK), and lactate dehydrogenase (LDH), strongly positive anti-nuclear antibodies human epithelial cell type-2 (ANA-HEp2), and a normal nerve conduction velocity test with precise MRI findings, a diagnosis of polymyositis was made. She was given bolus intravenous methylprednisolone for five days, followed by oral methylprednisolone with subcutaneous methotrexate weekly. She reported a 50% improvement in muscle weakness; however, partial bulbar weakness persisted at the time of discharge. On her next follow-up, her blood investigations for auto-antibodies were done. The autoantibodies anti-Jo-1 (3+), Ro-52 (2+), and Mi-2β (2+) were found to be positive. These investigations, coupled with the clinical features she was presenting, finally led us to conclude that it was a case of polymyositis complicated by the antisynthetase syndrome.

## Introduction

Antisynthetase syndrome and polymyositis are included in a broad group of heterogeneous diseases known as idiopathic inflammatory myopathies [1]. Polymyositis is an autoimmune disorder that presents with myalgia, swelling, tenderness, and proximal muscle weakness in the flexor muscles of the neck, pelvic region, thigh, and shoulders in the symmetric distribution. The estimated prevalence of polymyositis and dermatomyositis is five to 22 per 100,000 persons. The incidence of these diseases happens to be somewhere around 1.2 to 1.9 per million people per year. Though there is no official data for the epidemiology of antisynthetase syndrome, about a quarter of polymyositis/dermatomyositis cases may involve antisynthetase syndrome [2]. Dissecting the antisynthetase syndrome diagnosis, which complicates the presenting issue of polymyositis/dermatomyositis, is generally a herculean task for clinicians. The overall female-to-male incidence ratio is 2 to 3:1. In the United States, the African American to white ratio of incidence is 3 to 4:1. Antisynthetase syndrome includes the involvement of antibodies against aminoacyl transfer ribonucleic acid (tRNA) synthetase enzyme. Polymyositis patients experience difficulties performing repetitive movements, walking upstairs, working with their arms above their shoulders, or rising from a chair. Some patients may also have trouble swallowing due to throat muscles' weakness, leading to aspiration pneumonia. In rare cases, there is the involvement of respiratory muscles requiring mechanical ventilation. We present a case of polymyositis with generalized muscle weakness acute in onset and associated with significant bulbar and respiratory muscle involvement.

## Case presentation

We present the case of an 18-year-old lady who came to the outpatient department (OPD) of a public tertiary care hospital with myalgia along with proximal muscle weakness associated with the pelvic and pectoral girdle symmetrically for the last two and a half months. The weakness was preceded by febrile illness one week prior and was sudden in onset and progressed further. She was given a short course of oral steroids elsewhere, with which she had partial relief, but weakness reappeared after stopping the steroids. On admission, she was afebrile. In addition to having proximal myopathy, she admitted to having dysphagia upon deglutition of solid and liquid foods, dysphonia, and dyspnea (single breath count of only 14. There were no cutaneous rashes, fasciculations, tingling/ numbness, with no family history of autoimmune disorders. General physical examination revealed anasarca, while the neurological and sensory studies were normal. At a later stage, she had significant muscle weakness with no muscle wasting. Neurological examination showed intact higher mental function, and gag reflex was absent while the rest of the cranial nerves were normal. The sensory review was within the normal limits with no wasting of muscles. However, there was significant muscle weakness with grade 2/5 power at both the shoulder joints, 3/5 at elbows, wrists, hip joints, knee joints, and 4/5 at the ankle joints. All deep tendon reflexes were present, and there was bilateral plantar flexion.

There was no added sound on chest auscultation, and other systems findings were unremarkable. Her biochemical and hematological parameters are shown in Tables 1, 2. Viral screening for hepatitis B, C, and human immunodeficiency virus (HIV) was negative. Urine analysis revealed mild albuminuria without hematuria and pyuria. Indirect immunofluorescence assay was positive for antinuclear antibodies- human epithelial cells type-2 (ANA HEp2) with nuclear subtle speckled pattern and intensity 4+ (strongly positive) primary titer 1:100 and endpoint titer of 1:3200. Chest x-ray, echocardiogram (ECG), ultrasound abdomen, and pelvis were normal. The nerve conduction study was also normal. 

**Table 1:** Biochemical parameters of the patient at the time of hospitalization. RF: rheumatoid factor; LDH: lactate dehydrogenase; CPK-NAC: N-acetyl cysteine activated creatine phosphokinase

Parameter	Value	Reference range
Alanine transaminase	434 IU/L	<40
Aspartate transaminase	402 IU/L	<34
RF	5.9 IU/mL	0-20
CPK-NAC	10,215 IU/L	<145
Serum LDH	1,776 IU/L	<247
Serum urea	29 mg/dL	15-40
Serum creatinine	0.9 mg/dL	0.4-1.1
Serum bilirubin-total	0.8 mg/dL	0.2-1.2
Serum bilirubin-direct	0.4 mg/dL	0.1-0.4
Serum bilirubin-indirect	0.4 mg/dL	0.2-0.8
Serum Na^+^	147 mEq/L	135-147
Serum K^+^	4.7 mEq/L	3.5-5.5
Serum Ca^+2^	4.96 mEq/L	4.4-5.2
Total protein	5.7 g/dL	6.4-8.3
Serum albumin	3.3 g/dL	3.5-5

**Table 2:** Hematological parameters of the patient at the time of hospitalization. ESR: erythrocyte sedimentation rate; HBsAg: hepatitis B surface immunoglobulin; HCV: hepatitis C virus; ANA-Hep2: antinuclear antibodies-human epithelial cells type-2

Parameter	Value	Reference range
Hemoglobin	10.4 g/dlL	12-16.5 g/dlL
Total leucocyte count	11,000/mm^3^	4000-10000/mm^3^
Neutrophils	80%	40-60%
Lymphocytes	15%	20-40%
Red blood cell count	3.51 x10^6^/mm^3^	3.5-5.5 x10^6^/mm^3^
Platelets	270,000/mm^3^	150,000-450,000/mm^3^
ESR (Westergren)	45 mm/hour	Up to 20 mm/hour
HBsAg	Negative	
HCV	Negative	
HIV	Negative	
ANA-HEp2	Strongly Positive	

**Figure 1:**
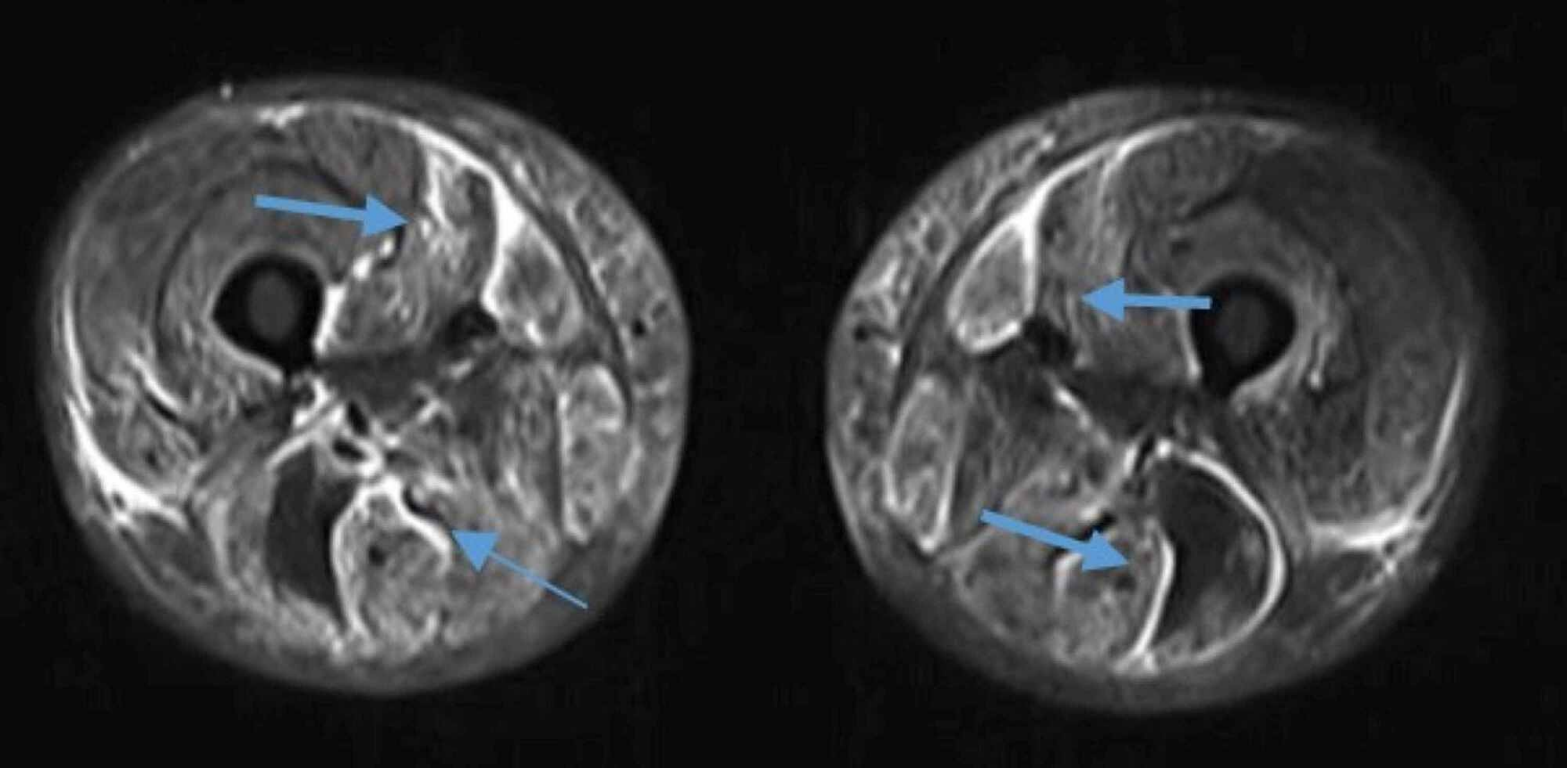
T2-weighted MRI thighs showing altered signals in thigh muscles (predominantly in adductors and extensors) symmetrically with circumferential involvement.

Magnetic resonance imaging (MRI) of the thigh was highly suggestive of myositis with symmetrical bilateral involvement (Figure 1). A pulmonary function test revealed a possible severe restriction, but the patient had inadequate respiratory efforts. Hence, we did high resolution computed tomography (HRCT) thorax, which showed no interstitial lung disease evidence. Based on proximal muscle weakness, elevated creatine phosphokinase (CPK), and lactate dehydrogenase (LDH), strongly positive ANA HEp2, and a normal nerve conduction velocity (NCV) with precise MRI findings, a polymyositis diagnosis was made. She was given bolus intravenous methylprednisolone 750 mg/day for five days followed by oral methylprednisolone 16 mg twice a day (0.8 mg/kg) with subcutaneous methotrexate 7.5 mg weekly.

Following this, she had a 50% improvement in muscle weakness; however, partial bulbar weakness persisted at the time of discharge. To that, she was asked to follow-up. When she showed up on the follow-up, her blood investigations for auto-antibodies were done. The autoantibodies anti-Jo-1 (3+), Ro-52 (2+), and Mi-2β (2+) were found to be positive. Based on these investigations conjugated with the presenting clinical features, the diagnostic criteria for polymyositis complicated by antisynthetase syndrome was fulfilled. She was then started on the following drug regimen (Table 3).

**Table 3:** The drug regimen administered to the patient. Note: SOS, if necessary.

Serial number	Dosage form	Drug	Titration
1.	Injection (subcutaneous)	Methotrexate	7.5 mg once a week
2.	Tablet	Folic Acid	5 mg once a day except on the day when methotrexate was administered
3.	Tablet	Methylprednisolone	32 mg once a day
4.	Tablet	Vit. D3	60,000 IU once a month
5.	Tablet	Pantoprazole	40 mg once a week
6.	Tablet	Paracetamol	500 mg SOS

## Discussion

Antisynthetase syndrome is an autoimmune condition that manifests clinically through signs and symptoms, such as interstitial lung disease, myositis, Raynaud’s phenomenon, fever, hyperkeratotic fingertips (mechanic’s hands), and arthritis (Table 4) [3]. It is associated with antibodies against aminoacyl tRNA synthetase enzyme, the most common antibody being the anti-Jo-1 [4]. Other antibodies that are commonly associated with the antisynthetase syndrome include anti-PL-7 and anti-PL-12. It is rarely diagnosed in general clinical practice, which adds to the dubiousness involved in its treatment. It has a typical constellation of symptoms in up to 30% of patients with polymyositis [5]. Polymyositis is an autoimmune inflammatory myopathy that presents as proximal myopathy and multisystem involvement, including respiratory, cardiac, and gastrointestinal systems. Some cases may have significant respiratory and bulbar participation, leading to aspiration pneumonia and respiratory failure. Evaluation of other systems is also necessary to correctly monitor the disease course. Polymyositis, complicated by antisynthetase syndrome, usually involves lung manifestations that affect the prognosis. In this case report, we present the case of a lady who got diagnosed with polymyositis complicated by the antisynthetase syndrome.

**Table 4:** Proposed diagnostic criteria for the antisynthetase syndrome.

Proposed diagnostic criteria for antisynthetase syndrome by Connors et al. (2010) [2]
Required: Presence of autoantibodies against amino-acyl tRNA synthetase enzyme
Plus one or more of the following clinical features: Raynaud’s phenomenon, arthritis, interstitial lung disease, fever (unattributable to other causes), mechanic’s hands (hyperkeratotic fingertips)

Polymyositis complicated with the antisynthetase syndrome is associated with multisystem dysfunction. Inflammation of the lung tissue leads to interstitial lung disease development in a vast population of individuals with polymyositis complicated by the antisynthetase syndrome. Respiratory muscle weakness results in restrictive lung disease, rarely pharyngeal, and upper esophageal muscles weakness, leading to dysphagia, which further aggravates because of the weakness of neck region muscles, resulting in an increased risk of aspiration pneumonia. Previous studies show that it is present in up to 70% of patients when investigated with sensitive techniques, such as HRCT, pulmonary function tests, and diffusion capacity.

Cardiovascular involvement is a risk factor for death among patients with polymyositis. Cardiac conduction abnormality, myocarditis can be associated with polymyositis. However, clinically evident heart involvement is rare; therefore, cardiac evaluation with ECG, echocardiography, Troponin-I is required. Constipation, diarrhea, stomach pain, gastric reflux is due to disturbed motility of the gut tract inflammation. Polymyositis can be associated with malignancies, including hematologic malignancies, such as lymphoma, solid tumors such as lung, ovarian, breast, and colon cancer. The screening for malignancies should include, at a minimum, a careful clinical examination, routine blood tests, and a chest radiograph. For women, mammography and a gynecologic study should be conducted as well. Our patient did not show any evidence of malignancy, cardiac involvement. The patient has severe weakness in respiratory muscles, pharyngeal muscles, and upper esophageal muscles leading to dysphagia for solid and liquid foods.

Indeed, the addition of autoantibody profiles, characteristic histopathological and immunohistochemical features, and imaging techniques such as MRI would significantly strengthen the diagnosis and better define this disorder. However, our patient's diagnosis of the antisynthetase syndrome was made purely based on antisynthetase autoantibodies, certain clinical features, and MRI findings. Although these autoantibodies are present in approximately 20% of patients with polymyositis or dermatomyositis [6], a combination of MRI and P-31 magnetic resonance spectroscopy examination produces the most comprehensive and accurate evaluation of patient and outcome tool in the longitudinal analysis of response to therapy [7,8].

There is enough evidence from the literature and personal experience to leave no doubt that immunosuppressive drugs can be useful. There is currently inadequate data to suggest that anyone drug is superior to another, and the choice is primarily determined by personal experience, often from using the medicine to treat other diseases. Thus, rheumatologists tend to use methotrexate (up to 30 mg weekly), as they experience its use in arthritis and psoriasis, respectively. In contrast, many neurologists favor azathioprine (2.5 mg/kg body weight per day). Methotrexate can cause pneumonitis, and possibly this could be confused with the interstitial lung disease associated with myositis. Cyclosporin (up to 5 mg/kg body weight per day) has been advocated for use in childhood DM but is also used in the adult form of the disease. Mycophenolate mofetil (2 g daily) is currently in vogue. Cyclophosphamide has been given as intravenous pulses (up to 1 g/m2 body surface area) and oral treatment (up to 2 mg/kg body weight per day). There is some evidence suggesting that it is conducive to treating associated interstitial lung disease [9]. Immunosuppressive agents treat the pulmonary or muscle manifestations of the antisynthetase syndrome.

Our patient responded well upon administration of the drug regimen mentioned in Table 3, leading to improved weakness and dysphagia; however, she still has mild girdle weakness, and she is still recovering from the disease.

## Conclusions

Polymyositis may or may not be complicated by the antisynthetase syndrome. Treating patients under this dilemma is often a tough choice on part of the treating physician. We identified antisynthetase syndrome complicating an earlier diagnosed case of polymyositis in our patient. Corticosteroids and cytotoxic drugs are common therapies. The patient has responded well to the treatment and is now recovering from the disease.

## References

[1] Mathews MB, Reichlin M, Hughes G, Bernstein R (1984). Anti-threonyl-tRNA synthetase, a second myositis-related autoantibody. J Exp Med.

[2] Cheeti A, Brent LH, Panginikkod S (2020). Autoimmune myopathies. StatPearls.

[3] Connors GR, Christopher-Stine L, Oddis CV, Danoff SK (2010). Interstitial lung disease associated with the idiopathic inflammatory myopathies: what progress has been made in the past 35 years?. Chest.

[4] Mathews MB, Bernstein RM (1983). Myositis autoantibody inhibits histidyl-tRNA synthetase: a model for autoimmunity. Nature.

[5] Muthusamy P (2020). Myopathy. http://www.clevelandclinicmeded.com/medicalpubs/diseasemanagement/neurology/myopathy/.

[6] Park JH, Vital TL, Ryder NM (2001). Autoantibody profiles in the sera of european patients with myositis. Ann Rheum Dis.

[7] Park JH, Vital TL, Ryder NM, Hernanz-Schulman M, Partain CL, Price RR, Olsen NJ (1994). Magnetic resonance imaging and P-31 magnetic resonance spectroscopy provide unique quantitative data useful in the longitudinal management of patients with dermatomyositis. Arthritis Rheum.

[8] Andersson H, Kirkhus E, Garen T, Walle-Hansen R, Merckoll E, Molberg Ø (2017). Comparative analyses of muscle mri and muscular function in anti- synthetase syndrome patients and matched controls: a cross-sectional study. Arthritis Res Ther.

[9] Hilton-Jones D (2003). Diagnosis and treatment of inflammatory muscle diseases. J Neurol Neurosurg Psychiatry.

